# Neural crest cell recruitment and reprogramming as central drivers of embryonic limb regeneration

**DOI:** 10.1073/pnas.2519994122

**Published:** 2025-12-23

**Authors:** Béryl Laplace-Builhé, Gautier Tejedor, Jholy De La Cruz, Audrey Barthelaix, Frédéric Marmigère, Dora Sapède, Sarah Bahraoui, Lucie Diouloufet, Stéphanie Ventéo, Jérôme Collignon, Christian Jorgensen, Farida Djouad

**Affiliations:** ^a^Institute for Regenerative Medicine and Biotherapies, University of Montpellier, INSERM, Montpellier 34295, France; ^b^Institute of Functional Genomics of Lyon, CNRS, École Normale Supérieure, Lyon 69007, France; ^c^Institute of Neurosciences of Montpellier, University of Montpellier, INSERM, Montpellier 34295, France; ^d^Université Paris Cité, CNRS, Institut Jacques Monod, Paris F-75013, France; ^e^University Hospital Center Montpellier, Montpellier 34295, France

**Keywords:** regeneration, neural crest cell, mouse embryo

## Abstract

Unlike regeneration-competent species, mammals lack epimorphic regeneration capacity, except for the most distal part of their digits. Here, we show that E10.5 mouse embryos can initiate regeneration of their forelimb bud (FB), but this capacity is lost by E12.5. Using comparative transcriptomics and in vivo lineage tracing approaches in the mouse embryo, we were able to identify a population of neural crest–derived cells (NCdCs) reexpressing early NC lineage molecular markers, *Wnt1* and *Foxd3*, specifically associated with regeneration at E10.5. Functional studies further reveal that these cells are required for FB regeneration and that the regenerative capacity lost in limb buds lacking NCdCs can be restored by exogenous transplantation of neural crest cells at E10.5. This work provides valuable information on the potential and prerequisites for regeneration in mammals.

## Significance

Mouse embryos possess the remarkable ability to regenerate amputated forelimb buds at E10.5—a capacity lost just 2 d later. We identify neural crest cells (NCCs) as key drivers in this transient regeneration. After limb amputation, NCCs rapidly accumulate at the injury site and reexpress early lineage genes; removal of these cells or silencing of these genes prevents regeneration. Strikingly, regeneration can be rescued by transplanting NCCs. These findings reveal the role of NCCs in embryonic limb bud regeneration and open new possibilities for unlocking regenerative potential in nonregenerative tissues.

Certain species such as salamanders and newts can fully regenerate a lost or damaged limb, restoring both its original structure and function through a process called epimorphosis (1). Epimorphic regeneration relies on the formation of a highly proliferative structure called a blastema, composed of a heterogeneous population of lineage-restricted cells that can contribute to their tissue of origin during the regeneration process (2, 3). In contrast to such regeneration-competent species, adult mammals exhibit no epimorphic regenerative capacity, except for the most distal part of their digits (4–6). A pioneering study has shown that E10.5 mouse embryos can initiate the regrowth of limb bud–like tissue after forelimb bud (FB) amputation (7). However, the cellular and molecular characteristics of the regenerating tissue have not been investigated, and the underlying mechanisms remain unknown. Here, we show that at E10.5, early regeneration response occurs through the transient formation of a blastema, a capacity lost by E12.5. Using comparative transcriptomics alongside lineage tracing experiments, we identified a neural crest–derived cell population specifically linked to regenerative activity at this stage. Functional analysis demonstrated that these cells were essential for blastema formation and tissue regeneration and that their absence abolished mouse embryo regenerative competence. Exogenous grafting of neural crest cells (NCCs) at E10.5 restored regeneration in NCdC-depleted FB, indicating a direct and stage-specific requirement for this population. This work provides insights into the potential and prerequisites for regeneration in mammals. Moreover, the innovative methodology we developed offers a powerful approach to uncover therapeutic targets and to evaluate strategies aimed at unlocking regenerative capacity across diverse tissue injuries and degenerative diseases.

## Results

### Regenerative Capacity of Mouse Embryo FB: Present at E10.5 but Lost by E12.5.

**Fig. 1.**
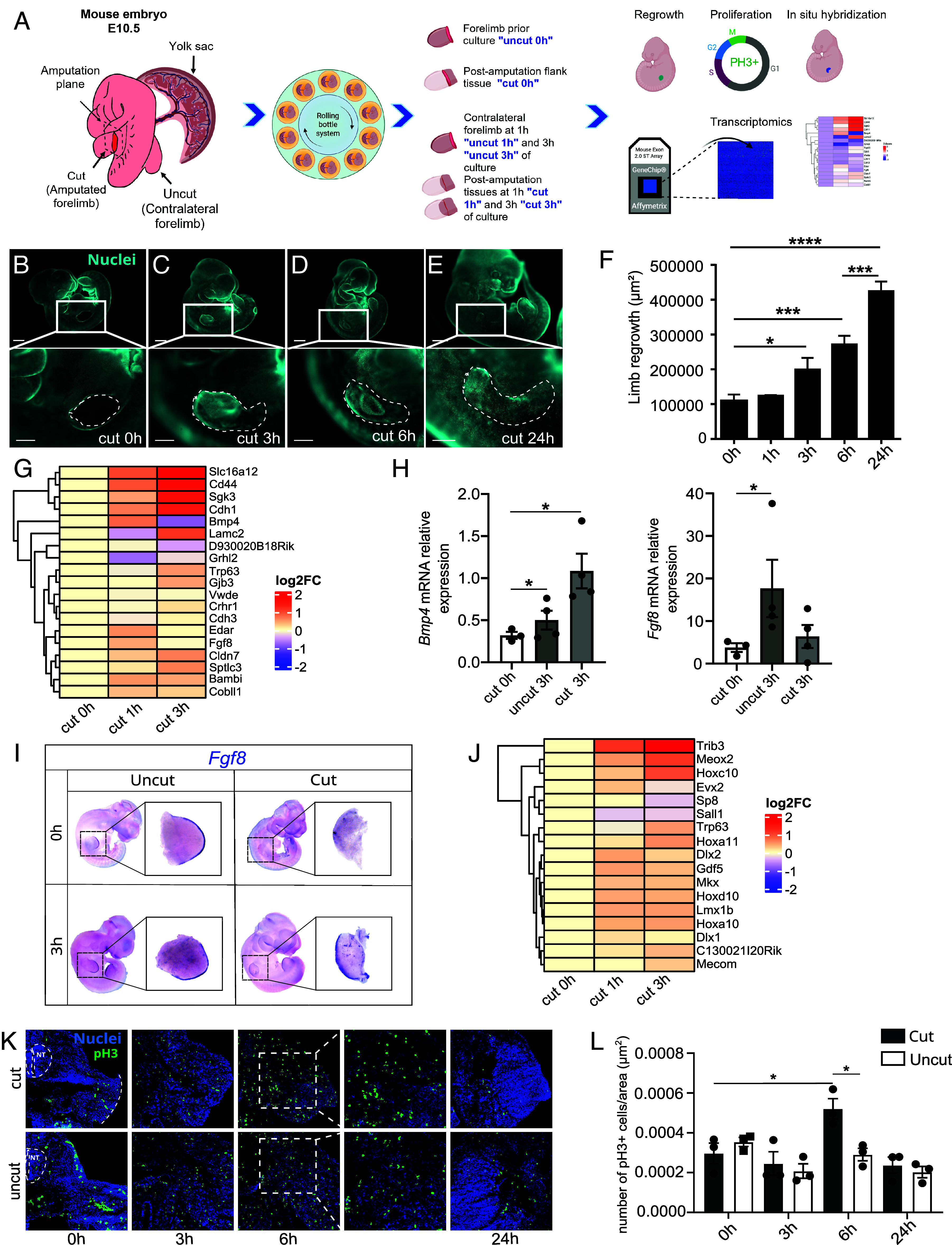
Assessment of mouse embryos FB regeneration at E10.5. (*A*) Experimental workflow and sample annotation. (*B*–*E*) Images of E10.5 embryos, cultured, fixed, and labeled with a fluorescent marker at 0, 3, 6, and 24 h after amputation (for the *Upper* panels scale bar, 800 µm, for the *Bottom* panels scale bar, 400 µm). Dashed lines delineate the area measured for quantification of regrowth. (*F*) Measurements of the area of limb regrowth 0, 1, 6, and 24 h after E10.5 FB amputation. Error bars are SEM (n = 2 to 9 embryos per group from at least two independent experiments, *P < 0.05, ***P < 0.001, ****P < 0.0001). (*G*) Microarray expression profiles of AER markers in postamputation tissues of E10.5 embryos at 0 hpA (cut 0 h) and in regenerating tissues at 1 hpA (cut 1 h) and 3 hpA (cut 3 h) normalized to cut 0 h. (*H*) Relative expression of *Bmp4* and *Fgf8* mRNA assessed by RT-qPCR in the tissues surrounding the amputation area of E10.5 mouse embryos (cut 0 h), the FB of ex utero developing intact E10.5 mouse embryos (3 h postculture/uncut 3 h) and the blastema of ex utero regenerating E10.5 mouse embryos (3 hpA/cut 3 h). (*I*) In situ hybridization against *Fgf8* mRNA in E10.5 mouse embryos. All in situ hybridizations were performed with E10.5 embryos before and after 3 h of culture. In intact (contralateral) E10.5 FBs, *Fgf8* expression is restricted to the AER, and this expression is maintained after 3 h of culture. Some *Fgf8*-positive cells were also found within the limb bud. In amputated embryos, ablation of FB eliminates the AER and most *Fgf8*-positive cells. After 3 h, *Fgf8*-positive cells were observed de novo in a structure resembling the AER of the contralateral side. A strong *Fgf8* signal was also seen in cells populating the regenerating FB. (*J*) Expression profile of distal mesenchymal markers in postamputation flanking tissues of E10.5 embryos at 0 hpa (cut 0 h) and in regenerating tissues 1 hpa (cut 1h) and 3 hpa (cut 3 h). (*K*) Proliferation was assessed by immunofluorescence using a pH3 antibody (green) on cryosections of E10.5 FBs at 0 h, 3 h, 6 h, 24 h postamputation, counterstained with Hoechst (blue) (NT is neural tube, the dashed line is amputation site). (*L*) Quantification of pH3-positive nuclei on cryosections such as those shown in (*K*) revealed an increase in proliferation in regenerating E10.5 FBs 6 hpA (Graphs represent means, error bars are SEM).

The limited understanding of limb regeneration in mammals is partly due to the challenges of performing amputations and monitoring subsequent events in utero. To study the regeneration of the FB in mouse embryos, we therefore used the roller culture system, which can support mouse embryo development ex vivo at relevant stages for 24 to 48 h (8, 9). As a first step, we performed basal excision of the FB in E10.5 embryos and observed the development of an outgrowth at 24 hours postamputation (hpA) (Fig. 1 *A*–*E*). This outgrowth, referring to the regrowing limb and extending from the proximal region to the newly forming structure, initiates between 1 and 3 hpA and continues up to 24 hpA (Fig. 1*F*). At the distal tip of the developing FB, the apical ectodermal ridge (AER) (10–12), a temporary structure present in all vertebrates, acts as a signaling center that maintains proliferation and survival of the underlying mesodermal cells. To investigate whether the newly formed tissue displayed an AER-like molecular identity, we examined the expression and localization of canonical AER marker genes. First, we used microarrays to examine the expression profile of a set of AER markers identified from a meta-analysis of publicly accessible single-cell data (13). Comparing the regenerating tissues at 1 and 3 hpA (cut 1 h and cut 3 h) with postamputation flanking tissue (cut 0 h) from E10.5 embryos revealed that a large panel of AER-associated genes was either transiently or more stably upregulated in the regenerating tissue (Fig. 1*G*). We further focused on *Bmp4* and *Fgf8*, which were transiently upregulated after amputation. Both encode factors secreted by the AER, with FGF8 regulating limb proximo-distal (PD) patterning and growth and BMP4 controlling AER induction and maturation (14, 15). Consistent with the microarray data, RT-qPCR analysis of the regenerated tissue at 3 hpA (cut 3 h) showed an increase in *Bmp4* and *Fgf8* mRNA expression levels compared to postamputation flanking tissue (cut 0 h) (Fig. 1*H*). However, this increase was significant only for *Bmp4*, likely due to the modest and/or locally restricted rise in *Fgf8* expression, which might become diluted as the blastema grows, potentially explaining the transient elevation of these markers. To explore that hypothesis, we assessed *Fgf8* expression by in situ hybridization and detected it at the distal border of 3hpA regenerating limb buds, in a pattern similar to the AER of intact FBs (Fig. 1*I*). Then, we further evaluated the expression profiles of genes involved in PD patterning during FB development, including *Hoxa10, Hoxc10, Hoxd10,* and *Hoxa11*, and observed a transient or more stable induction of all these genes in 1 and 3 hpA regenerating FBs (Fig. 1*J*), suggesting the emergence of a distal identity within the forming tissue. Finally, we assessed the proliferation rate in developing vs. regenerating FB tissues of E10.5 mouse embryos by phosphorylated Histone 3 (pH3) immunodetection (Fig. 1*K*). We observed a sustained cell proliferation at 3, 6, and 24 hpA in both amputated and intact (control) conditions, as the FB is still developing at this stage. However, at 6 hpA, a significant increase in proliferation was observed in regenerating FBs compared to the postamputation tissue (cut 0 h) (Fig. 1*L*). Similar analyses performed in E12.5 embryos revealed a lack of regenerative capacity of the FB, including a lack of tissue regrowth at 24 hpA (*SI Appendix*, Fig. S1 *A*–*F*), no induction of AER-associated markers (*SI Appendix*, Fig. S1 *G*–*I*), and a lack of expression of distal genes (*SI Appendix*, Fig. S1*J*). In line with these results, cells in both amputated and intact FBs of E12.5 embryos exhibited similar levels of proliferation, as indicated by the presence of pH3-positive cells, with no significant difference observed between the two conditions at all stages (*SI Appendix*, Fig. S1 *K* and *L*).

Collectively, these results show that in E10.5 embryos, FB amputation is followed by a rapid and transient increase in cell proliferation, accompanied by an induction of AER markers at the distal tip of the regenerating tissue, all consistent with the formation of a functional blastema, whereas E12.5 FBs show no sign of regeneration.

### FB Regeneration in E10.5 Mouse Embryos Is Associated with Local Activation of NCC Genetic Programs.

**Fig. 2.**
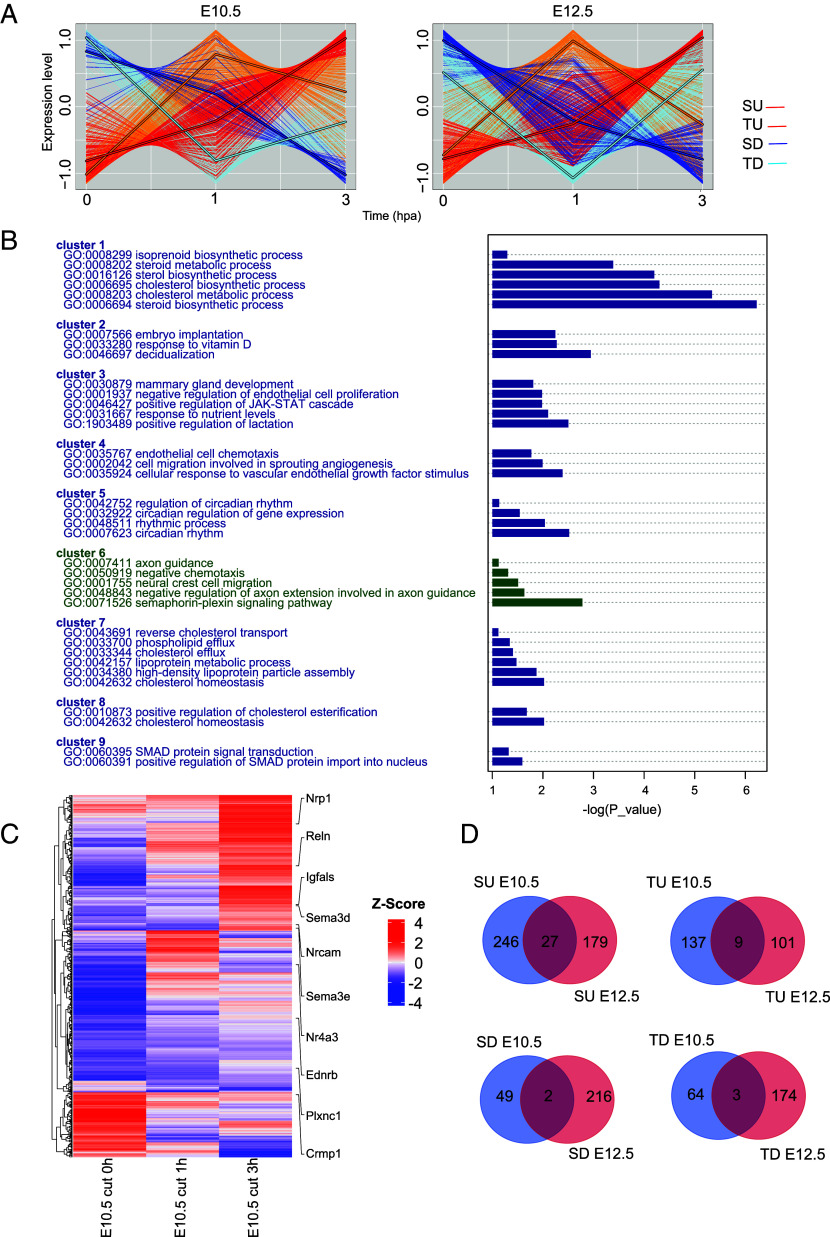
Differential gene expression kinetics of postamputation tissues at E10.5 and E12.5 stages. (*A*) Clustering of DEGs after E10.5 or E12.5 FB amputation according to whether they are transiently up- or down-regulated, or steadily up- or down-regulated. (*B*) Functional clustering of GO terms specifically enriched in DEGs from E10.5 postamputation tissues (NCC migration cluster 6 is highlighted in green). (*C*) Heatmap of DEGs in E10.5 postamputation tissues with annotation of NCC migration-associated genes. (*D*) Comparative analysis of the composition of the gene clusters differentially regulated postamputation of E10.5 and E12.5 FBs. SU: Steady upregulation; TU: transient upregulation, SD: Steady downregulation and TD: Transient downregulation.

To identify the cellular and molecular events underlying the stage-specific amputation response, we compared the dynamics of differentially expressed genes (DEGs) during the 3 h following amputation in E10.5 and E12.5 embryos. During the first 3 hpA, DEGs could be clustered into four distinct expression patterns: i) steady upregulation (SU), ii) transient upregulation (TU, highest expression at 1 hpA), iii) steady downregulation (SD), and iv) transient downregulation (TD, lowest expression at 1 hpA) (Fig. 2*A*). Gene ontology (GO) clustering of enriched GO terms in the E10.5 embryos identified gene groups associated with distinct biological functions. Notably, cluster 6 was identified, encompassing genes associated with neural crest cell (NCC) migration (GO:0001755) (Fig. 2*B* and *SI Appendix*, Fig. S2*A*). In this cluster, we specifically identified genes expressed in NCCs/neural crest–derived cells (NCdCs) such as *Ednrb* and *Reln,* and genes of the *semaphorin-plexin* pathway (GO:0071526), one of the main NCC migration-associated pathways, such as *Nrp1, Plxnc1, Sema3c, Sema3d,* and *Sema3e* (Fig. 2*C*). These genes were mainly found in the SU cluster, suggesting an accumulation of migrating NCCs within the blastema. Regarding E12.5-specific gene clusters, we identified an enrichment of GO terms related to inflammation, chemotaxis, and cytokine production, indicating a robust immune response following FB amputation that might be related to a repressed regenerative response at this stage (16) (*SI Appendix*, Fig. S2*B*). Of note, only a limited set of DEGs was shared between E10.5 and E12.5 postamputation tissues across the four groups. Specifically, 6%, 2%, 0.7%, and 1% of the expressed genes were shared between the two stages in the SU, TU, SD, and TD groups, respectively (Fig. 2*D*), underscoring the unique and stage-specific transcriptomic responses to amputation at each embryonic stage.

Altogether, these results correlate the FB regeneration process in E10.5 mouse embryos with local activation of genetic programs associated with NCC/NCdC identity and migration.

### NCdCs Reexpressing Early-Developmental Stage NC Markers Accumulate in the Regenerating FBs of E10.5 Embryos.

**Fig. 3.**
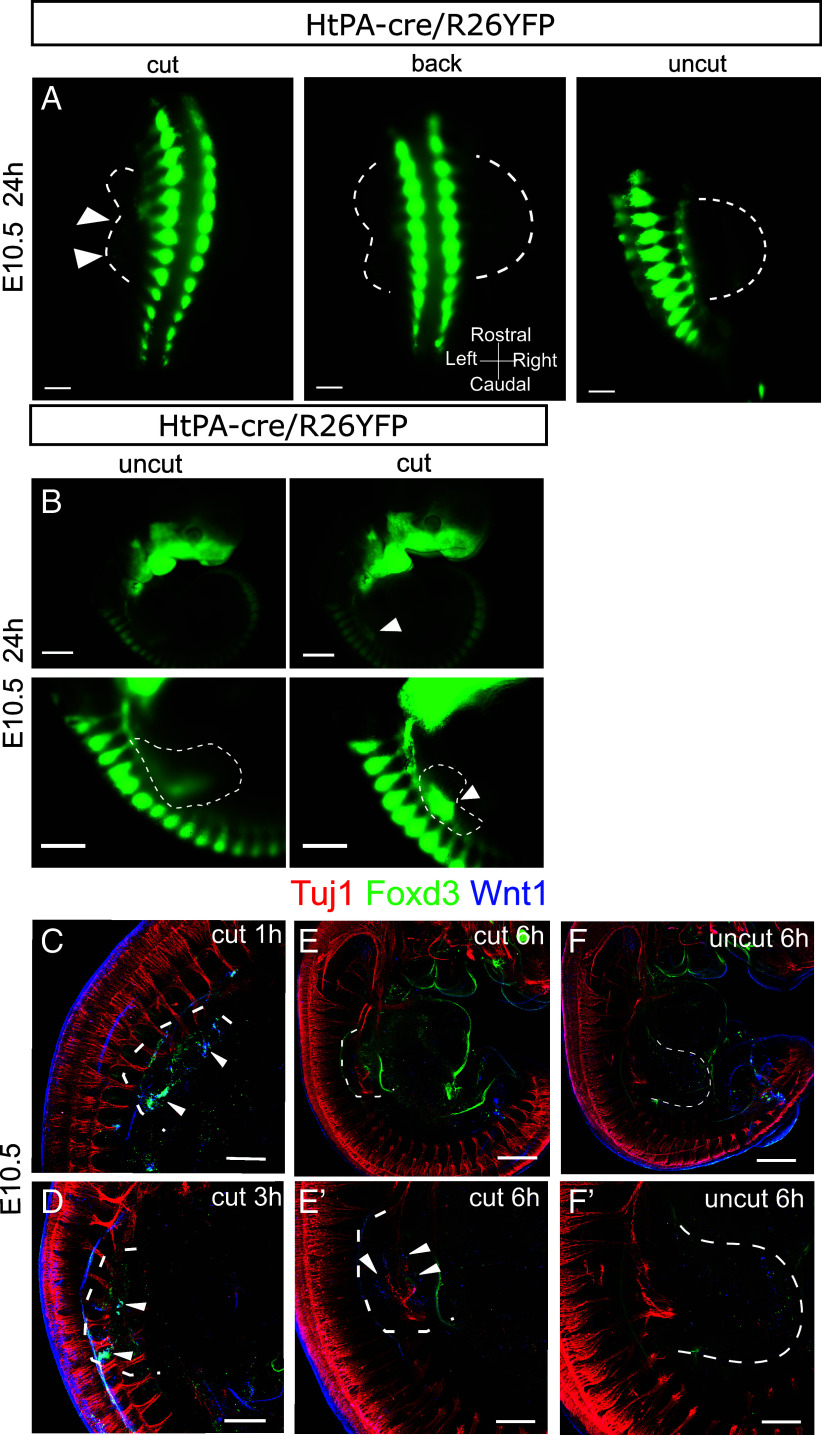
NCdC are recruited to the amputation site at E10.5 and express Wnt1 and Foxd3. (*A* and *B*) Macroscopic images showing amputated or contralateral limbs of HtPA-cre/R26YFP, E10.5 embryos 24 hpA. NCdCs are green (indicated by white arrowheads in the regenerating FB). (*A*) Views from the back of a HtPA-cre/ R26YFP E10.5 embryo at 24 hpA (Scale bar, 800 µm; n = 3). (*B*) Lateral views of HtPA-cre/R26YFP E10.5 embryos, both nonamputated (uncut) and amputated (cut), at 24 hpA. Scale bar: 400 µm for the top images and 800 µm for the bottom images. (*C*–*F’*) Images from confocal microscopy of cleared E10.5 embryos labeled with anti-bIII-TUBULIN (red), anti-WNT1 (blue), anti-FOXD3 (green) antibodies at 1 hpA (*C*), 3 hpA (*D*), and 6 hpA (*E* and *E’*). *F* and *F’* show the contralateral FB at 6 h postamputation (Scale bar for *C*, *D*, *E’*, *F’* = 400 µm for *E*, *F* = 200 µm). Dashed lines delineate the FB and the regenerating FB areas.

NCC delamination from the dorsal neural tube and migration to target destinations throughout the mouse embryo occurs from E8.5 to E10.5 (17, 18). NCCs express characteristic markers, notably WNT1, which is rapidly lost after migration, and FOXD3, whose expression is maintained in early migrating NCCs and then decreases in derivatives (19). To confirm the presence of NCCs/NCdCs in the blastema, we performed lineage tracing experiments of NCCs and their progeny using two independent NCC Cre-driving mice: the Wnt1-Cre and the PLAT-Cre transgenic lines (the latter hereafter referred to as HtPA-Cre), crossed with the R26-eYFP reporter mouse to generate Wnt1-cre/R26YFP or HtPA-cre/R26YFP E10.5 and E12.5 embryos. After FB amputation at E10.5, YFP+ cells accumulated in the blastema at 6 and 24 hpA (Fig. 3 *A* and *B* and *SI Appendix*, Fig. S3 *B* and *D*). In contrast, no YFP+ cells were found in the intact contralateral FBs (uncut 6 h) (*SI Appendix*, Fig. S3 *A* and *C*). Similar experiments were conducted with Wnt1-cre/R26βGal E10.5 embryos (*SI Appendix*, Fig. S3*E*). At 6 hpA, βGal-positive cells were detected distal to the dorsal root ganglion (DRG) on the amputated side of the embryo, but not on the uncut side (*SI Appendix*, Fig. S3*F*). Transverse cryosections of Wnt1-cre/R26βGal E10.5 embryos at 6 hpA revealed the presence of βGal-positive cells within the regenerating FBs.

At E12.5, FBs already contained YFP+ cells before amputation, but no further accumulation was observed upon wounding (*SI Appendix*, Fig. S3 *F* and *G*). The local accumulation of ectopic NCCs/NCdCs thus correlates with an environment permissive for regeneration.

We further examined whether ectopic NCCs/NCdCs in the regenerating FB correlated with the expression of characteristic NCC markers. Immunodetection revealed the presence of WNT1 and FOXD3 proteins in the regenerating FBs at 1 hpA (Fig. 3*C*), 3 hpA (Fig. 3*D*), and 6 hpA (Fig. 3 *E* and *E’*), consistent with the presence of NCCs/NCdCs observed within the regenerating tissue. In contrast, WNT1 and FOXD3 were not detected in the intact contralateral FBs of the same embryos at 6hpA (Fig. 3 *F* and *F’*), consistent with the absence of ectopic YFP+ cells in tracking experiments. The earlier detection of WNT1/FOXD3 by immunostaining compared to YFP/β-Gal in lineage tracing reflects the higher sensitivity of immunostaining, while Cre-mediated reporter activation requires additional time and sufficient accumulation of NCdCs to become detectable.

Since nerve-associated NCdCs have been shown to be required for the epimorphic regeneration of the digit tip in mouse (4), we investigated whether NCCs/NCdCs reexpressing WNT1 in the blastema were in contact with nerve fibers. Co-labeling with anti-TUJ1 antibody in E10.5 amputated mouse embryos indicated that cells expressing WNT1 and FOXD3 were in close proximity to the truncated nerve fibers at 1 hpA (Fig. 3*C*), 3 hpA (Fig. 3*D*) and 6 hpA (Fig. 3 *E* and *E’*). Of note, during the 6 h of culture, while we observed YFP^+^ cells both in the amputated and contralateral FBs of E12.5 embryos (*SI Appendix*, Fig. S3 *C* and *D*), we found no expression of WNT1 and FOXD3, suggesting that at this stage, NCC-derivatives are not able to induce early NCC markers upon amputation (*SI Appendix*, Fig. S3 *H* and *I*). Altogether, these results strongly suggest that in contrast to the E12.5 stage, NCCs/NCdCs accumulate at the wound site at E10.5 and are able to reexpress early NC markers.

### NCCs/NCdCs Reexpressing Early-Developmental Stage NC Markers Are Necessary for FB Regeneration in E10.5 Mouse Embryos.

**Fig. 4.**
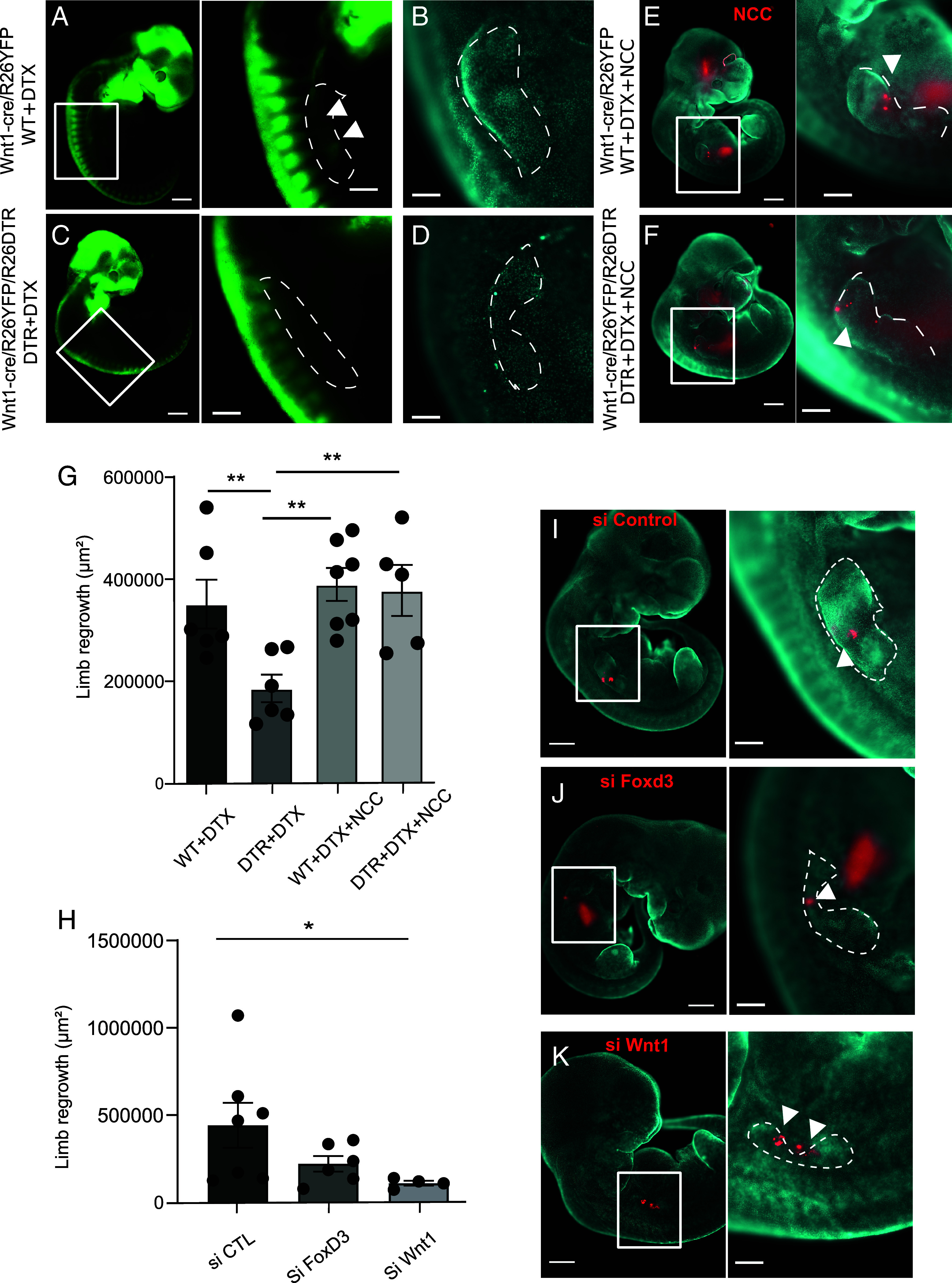
Neural crest-derived cells expressing *Foxd3* and *Wnt1* are necessary to promote forelimb regeneration in E10.5 embryos. (*A*–*D*) Macroscopic images of E10.5 Wnt1-cre/R26YFP embryos (*A* and *B*) and E10.5 Wnt1-cre/R26YFP/R26DTR embryos injected with DTX in the forelimb (*C* and *D*), 24 h after amputation. YFP+ neural crest–derived cells are in green (*A* and *C*), nuclei stained with Hoechst are in cyan (*B* and *D*), white arrowheads indicate YFP+ NCdC recruitment in (*A*) (Scale bar, 400 µm). (*E* and *F*) Macroscopic images showing E10.5 Wnt1-cre/R26YFP embryos (*E*), and E10.5 Wnt1-cre/R26YFP/R26DTR embryos injected with DTX and Dil labeled neural crest cells (in red, white arrowheads) (*F*), 24 h after amputation, and corresponding close-ups of their FBs. (*G*) Corresponding graph of FB area quantification at 24 h in indicated conditions (error bars are SEM, n = 5 embryos per condition from at least 2 different experiments, **P < 0.01). *(H)* Corresponding quantification of FB area at 24 h for the conditions shown in *(I)*, *(J)*, and *(K)* (Error bars are SEM, *P < 0.05). Macroscopic images of E10,5 embryos and their FBs at 24 h postamputation, injected with siRNAs [stained in red, control siRNA, (*I*), *Foxd3* siRNA (*J*), *Wnt1* siRNA (*K*)] and stained with Hoechst (Scale bar, 800 µm, and 200 µm for the forelimb images). Dashed lines delineate the area measured for quantification of regrowth.

We then investigated the role of NCCs/NCdCs in the FB regeneration process. We took advantage of the R26DTR mouse that carries the diphtheria toxin receptor (DTR) allele and crossed it with the Wnt1-cre mouse. E10.5 Wnt1-cre/R26YFP/R26DTR mouse embryos were amputated and injected with diphtheria toxin (DTX) at the amputation site to locally deplete Wnt1-cre-expressing cells and thus block their accumulation there for up to 24 hpA (Fig. 4 *A* and *C*). Depletion of these cells completely abolished FB regeneration in E10.5 amputated embryos (Fig. 4 *B*, *D*, and *G*). To confirm that FB regeneration requires a NCC-like population, we asked whether a graft of exogeneous E10.5 NCCs into DTR embryos treated with DTX could rescue the regenerative potential. We isolated and cultured YFP^+^ NCCs obtained from E10.5 Wnt1-cre/R26YFP embryos (20). We then transplanted these E10.5 NCCs stained with CM-DiI to the amputation site of locally NCCs-depleted E10.5 embryos. We found that exogeneous NCC transplantation did rescue FB regeneration when Wnt1-cre-expressing cells had been locally depleted (Fig. 4 *E*–*G*). Of note, we confirmed that DTX-treated E10.5 WT embryos regenerated as well as untreated controls, either in the presence or absence of exogenous NCC grafts (Fig. 4*G*). Since NCdCs only accumulated in E10.5 FBs in response to amputation, concomitant with WNT1 and FOXD3 expression, we asked whether the function of these genes was required for FB regeneration. We developed a method to locally silence *Foxd3* and *Wnt1* using a lipid-based in vivo transfection reagent, Injectin, mixed with small interfering RNAs (siRNAs) directed against *Foxd3* and *Wnt1* mRNAs. This solution was injected into 4 different sites in the FB amputation area of E10.5 embryos and induced a significant decrease in the expression level of these two genes at 24 hpA (*SI Appendix*, Fig. S4 *A* and *B*). We then tested the effect of reducing *Foxd3* or *Wnt1* expression on FB regeneration. Amputated E10.5 embryos were injected with siRNAs prior to being cultured in the rolling system for 24 h. FB regrowth, assessed by measuring the area from the transected plane to its most proximal end, was significantly impaired after si*Foxd3* or si*Wnt1* injection (Fig. 4 *H*, *J*, and *K*) compared to the control condition (Fig. 4 *H* and *I*). Taken together, these results demonstrate that NCdCs reexpressing *Wnt1* and *Foxd3* are required for FB regeneration in E10.5 mouse embryos. While our findings strongly support a regeneration-specific role, we cannot exclude additional contributions of these factors to other processes; further experiments would be needed to formally address their role in limb development.

## Discussion

This study provides insights into the process of limb bud regeneration in the mammalian embryo. First, our findings reveal that this regenerative capacity is confined to a limited developmental time window and rapidly lost in mouse embryos. Although the FB regrowth potential at E10 has been suggested based on the formation of an outgrowth (7) after amputation, this study investigates the process following FB amputation at the cellular and molecular levels and to compare the two states (regenerative and nonregenerative). Moreover, we investigate here the underlying phenomenon of the postamputation outgrowth, focusing on key events that define the regeneration process, such as the formation of the AER, blastema cell proliferation, and the gene expression pattern involved in PD patterning during FB development.

Furthermore, our results demonstrate that the unique capacity of NCdCs to mobilize and to reinduce the expression of markers characteristic of a less differentiated stage in response to an injury is essential to orchestrate FB regeneration. The reexpression of WNT1 and FOXD3 in the regenerating FB represents a significant advancement in our understanding of the molecular programs associated with regeneration and suggests that the differentiation state of NCdCs may contribute to this process. While these markers are crucial during NC embryonic development, their reactivation in NCdC has never been described and investigated during regeneration. Our findings reveal that although NCdCs are present in adult mammals, their capacity to revert to an early, transient developmental state is restricted, hindering effective regeneration. Consequently, limb bud regeneration in mammals would depend on a highly coordinated cellular and molecular program that is only transiently inducible. The inability of adult NCdCs to fully dedifferentiate underscores the challenges in harnessing regenerative mechanisms observed during embryogenesis.

Interestingly, the role of NCdCs, particularly Schwann cells present in adult vertebrates, has also been documented in epimorphic regeneration, as observed in the limbs of newts (21) and in the digit tips of mammals (4). Our study therefore provides additional evidence that NCdCs are at the hub of the regenerative responses to amputation across vertebrate evolution.

Because our ex vivo embryo culture system cannot be maintained beyond 24 h, we cannot determine whether a fully patterned limb would eventually be restored. For this reason, we describe here the early phase of regeneration, corresponding to the regrowth of a limb bud–like structure. This limitation is intrinsic to the culture system rather than the tissue’s regenerative potential. Nonetheless, the formation of a blastema, active proliferation, and reactivation of developmental patterning genes are consistent with epimorphic regeneration.

In light of these findings, forelimb regeneration in the mouse embryo constitutes a valuable experimental model to investigate the mechanisms that enable regeneration, and to identify how these limitations could be overcome to promote this process at older ages and in pathological contexts for medical purposes.

## Materials and Methods

### Mouse Culture Embryo E10.5/E12.5 and Ablation.

All methods were carried out in accordance with relevant guidelines and regulations. All experimental protocols on animals were approved by the French Health Authorities (agreement no. B34-172-36) and in accordance with European and French Agricultural Ministry guidelines for the care and use of laboratory animals (Council directive 2010/63).

For the embryo culture experiments, we mated transgenic mice Wnt1-cre (22) or HtPA-cre (23) (gift from A. Pattyn, Institute for Neurosciences of Montpellier, University of Montpellier, INSERM, Montpellier, France) with ROSA26-YFP (provided from F. Constantini—JAX, Department of Genetics and Development, Columbia University, New York, USA) or double transgenic Wnt1-YFP and HTPA-YFP with ROSA26-DTR (gift from A. Moqrich—JAX, Aix Marseille Univ., CNRS, IBDM, Institut de Biologie du Développement de Marseille, Marseille, France). Mating was confirmed by checking for vaginal plugs the following morning, which was considered day 0.5 of fecundation (0.5 dpc). The pregnant mice were killed by cervical dislocation, before collecting E10.5 and E12.5 embryos. After collecting the embryo from the yolk sac, we performed an ablation on the left limb then put the embryos in a rolling bottle culture system. The procedure for embryo culture was conducted as previously described (8, 9).

### RNA Extraction, Array Hybridization, and Data Processing.

Total RNA was isolated from all samples using RNeasy Mini Kit (Qiagen). The amount and purity of extracted RNA were determined using a NanoDrop ND-1000 spectrophotometer (NanoDrop ND, Thermo Fisher Scientific) as well as their integrity by using the Agilent 2100 Bioanalyzer (Agilent Technologies; http://agilent.com/). cDNA generation, amplification, fragmentation, and biotinylation were performed using the Ambion WT Expression Kit (Ambion, Austin, TX, USA). Microarray experiments took place at the IRMB (Montpellier University Hospital) DNA microarray platform. All samples were hybridized to Affymetrix GeneChip® Mouse Exon 1.0 ST Arrays according to Affymetrix recommendations.

Tissues for microarray data generation consisted of three biological replicates from each condition [Flanking tissue (tf_0 hpA), amputated tissues at 1- and 3-hpA, and the contralateral intact FB at 0, 1, and 3 h of postembryo culture were collected for both embryonic stages, E10.5 and E12.5]. Data were acquired on a GeneChip® Scanner 3000 and CEL file generation was performed using AGCC.

### Microarray Analysis.

CEL files were processed and analyzed in Rstudio following the Klaus and Reisenauer analytical workflow (24). Probe intensity data were extracted, normalized, and summarized at the gene level with Robust Multi-chip Average (RMA) method from oligo package.

Meta-analysis of public single-cell data for distal mesenchyme and AER markers (13) was performed to extract specific markers for AER and Distal mesenchyme populations using Seurat package FindMarkers function in Rstudio which average expression from triplicates was then used for heatmap representation. Gene expression kinetics analysis was performed by determining the DEGs in pairwise comparisons between samples (TF, 1, 3 hpA) within each developmental stage. Differential gene expression analysis was performed by building a linear model using limma package. Genes were considered as differentially expressed (DEG) if they displayed a *P*-value < 0.01 and an absolute value of logarithm foldchange higher than 0.5. DEGs were then clustered using k-means clustering algorithm to investigate postamputation kinetics. DEGs were then used for functional clustering of gene ontology terms following DAVID annotation database guidelines (25) retaining terms with an enrichment *P*-value < 0.05. Finally, stage-specific terms were used for graphical representation.

All our data are accessible at the Gene Expression Omnibus (GEO) repository (https://www.ncbi.nlm.nih.gov/geo/) with the provisional accession series number GSE292836.

### RT-qPCR Analysis.

RNA extraction was performed using RNeasy mini kit (Qiagen S.A.). A total amount of 500 ng of RNA was used to reverse transcript using the Multiscribe reverse transcriptase (Applied Biosystems). Quantitative PCR was performed using the SYBR Green I Master kit and a LightCycler® 480 Detection system, following the manufacturer’s recommendations (Roche Applied Science). The following primers were used: RPS9-Forward: GCTGTTGACGCTAGACGAGA—RPS9-Reverse: ATCTTCAGGCCCAGGATGTA—Wnt1-Forward: CGAGAGTGCAAATGGCAATTCCG—Wnt1-Reverse: GATGAACGCTGTTTCTCGGCAG—FoxD3-Forward: CAAGAACAGCCTGGTGAAGCCA—FoxD3-Reverse: AGGGTTGCTGATGAACTCGCA.

### Diphteria-Toxin Depletion/Neural Crest Cell Injection.

We used the recombination between transgenic Wnt1-cre-YFP mouse and Rosa26-Flx-Diphteria Toxin Receptor mice, to deplete the local Wnt1 derived cells after DTX (1 µg/mL—Sigma) injection. Neural crest cells were isolated from an E10.5 Wnt1-YFP embryo and injected in 4 different sites of 5 nL each in the amputated limb using the Eppendorf ® FemtoJet® microinjector.

### Whole-Mount In Situ Hybridization of Mouse Embryos.

Probe templates corresponding to the 400 last nucleotides of FGF8 (Mus musculus fibroblast growth factor 8, transcript variant 1) were obtained by PCR using the following primers Forward: AAG GCA AGG ACT GCG TAT TCA, Reverse: TAA TAC GAC TCA CTA TAG GGC CAA CAG CAA ACA ATA TGC AC and amplicons were flanked with T7 promoter. Amplicons were purified by MinElute Gel Extraction Kit (28604, Qiagen). Digoxigenin (DIG)-anti-sense RNA probes were synthesized by reverse transcription (T7 RNA Polymerase, EP0111 Thermo Fisher Scientific) using the DIG RNA labeling Kit (11 175 025 910, Roche) and purified on Illustra Microspin G50 columns (27533001, GE Healthcare, Chicago, IL). Then, in situ hybridization on whole-mount embryos was performed using the established protocol (26). E10.5 stage embryos were dissected in PBS, fixed overnight at 4 °C in 4% PFA-PBS, washed in PBS several times, progressively dehydrated in 100% EtOH and stored at 20 °C until use. Embryos were rehydrated gradually in PBS-0.1% Tween20 (PBT), bleached in 2% H2O2-PBT for half an hour, washed 2 times in PBT, treated with 10 µg/mL Proteinase K-PBT for 10 min, rinsed in PBT, and postfixed in 4% PFA-0.1% Glutaraldehyde-PBT for 20 min at 4 °C. Embryos were then washed in PBT, prehybridized in the hybridization buffer (50% Formamide-1.3xSSC-5 mM EDTA-50 µg/mL yeast tRNA-0.5% CHAPS-2% Tween 20) for 2 h at 70c, and hybridized overnight at 70 °C with the Dig-labeled FGF8 antisense-RNA probe diluted 1/100 in the hybridization buffer. Embryos were then washed two times 30 min in hybridization buffer, rinsed in TST (10 mM Tris pH 7.5, 0.5 M NaCl, 0.1% Tween 20), and incubated twice 30 min at 37 °C with 10 mg/mL RNase A-TST. They were then washed in TST at RT and then twice in hybridization buffer for 30 min at 65 °C. Embryos were then washed twice for 10 min and 1 h in MABT at RT, incubated in 20% Serum-MABT at RT for 1 h, and incubated overnight at 4 °C with the anti-DIG antibody (Roche) diluted 1/2,000 in 2% Serum-MABT. They were then washed in MABT for 3 d. They were rinsed several times in B3 and stained with the NBT/BCIP-B3 substrate solution (Roche). The staining was stopped by washing several times in PBT. Embryos were postfixed in 4% PFA-PBT, washed in PBT, progressively transferred in 80% Glycerol-PBT, and stored in the dark at 4 °C.

### Whole Mount Staining, Tissue Clearing, and Immunofluorescence.

The protocol for whole-mount immunofluorescence is adapted from ref. 27. The primary antibodies used for the labeling are GFP (Invitrogen A6455), Tub3 (Sigma T2200), Wnt1 (Invitrogen MA515544), and FoxD3 (Biotechne MAB5090). The secondary antibodies used are donkey anti-chicken AlexaFluor 488, donkey anti-mouse AlexaFluor 555, and donkey anti-rabbit AlexaFluor 594 (ABCAM).

For the clearing of embryos coupled with IF, the embryos are fixed overnight in 4% PFA at 4 °C and dehydrated in Methanol/PBS: 50% MeOH, 80% MeOH, 100% MeOH (15 to 30 min for each step with agitation and at room temperature) and then transferred to a solution of MeOH+6% H2O2 to bleach and incubated overnight at 4 °C and protected from light. Rehydrate in 100% MeOH x2, 80% MeOH (in PBS), 50% MeOH, PBS (15 to 30 min for each step with agitation and at RT). Incubate your sample in PBSGT (0.2% gelatin, 0.5% TritonX100 in PBS) for 2 d. Incubate with primary antibodies in PBSGT + 0.1% saponin (10 µg/mL) for 7 d at RT with rotation, then rinse in PBSGT, 6 times during 1 d at RT with rotation. For the secondary antibodies, incubate in PBSGT+ 0.1% saponin (10 µg/mL) at RT 70 rpm 2 d and then wash in PBSGT, 6 times during 1 d at RT with rotation. After this step, proceed to the tissue clearing with BABB method, starting with dehydrate with graded MeOH/PBT series: 25% MeOH, 50% MeOH, 75% MeOH, 2 × 100% MeOH (5 min for each step with agitation and at room temperature). After incubating the tissue twice for 5 min each in 50% MeOH–50% BABB (benzyl alcohol—Sigma B-1042 / benzyl benzoate—Sigma B-6630, 1:2 ratio), the tissue was incubated overnight in BABB solution at room temperature.

The pictures acquisition was performed on LSM 800 Confocal Microscope from Zeiss. LacZ staining was performed on embryos at 6 h postamputation fixed on ice for 20 min in Webster (2.5% glutaraldehyde and 0.5% formaldehyde in 0.1 M phosphate buffer, pH 7.3 to 7.4), following the protocol described in ref. 28. For immunofluorescence on NCC, we performed as follows: NCC were fixed with 4% PFA overnight at 4 °C. Then rinsed extensively with PBS, permeabilized with 0.1%triton in PBS, blocked with 1% donkey serum and 5% BSA in PBS. The primary antibodies were diluted in the blocking solution. The primary antibodies used for the labeling are GFP (Invitrogen A6455), WNT1 (Invitrogen MA515544), and FOXD3 (Biotechne MAB5090). The secondary antibodies used are donkey anti-chicken AlexaFluor 488, donkey anti-rat, and anti-mouse AlexaFluor 594 (ABCAM).

### Immunostaining on Cryosections.

Whole embryos were fixed overnight in PFA 4% in PBS 1× at 4 °C. They were transferred to a 25% sucrose bath for cryosection at 14 μm thickness and stored at −20 °C on slides to get comparable sections on each slide. Immunofluorescence was performed using a 1/500 dilution of a primary anti-pH3 and a 1/1,000 dilution of a secondary goat anti-mouse IgG—AlexaFluor 488 (Abcam).

### In Vivo siRNA Transfection.

The embryos were injected with 100 µM siRNA prepared in PBS. We decided to use the Injectin as a transfection agent and follow the recommendation provided by the manufacturer (BioCellChallenge). siRNAs were injected in the left forelimb excision zone. For the local injections of 5 nL each, we used the Eppendorf ® FemtoJet® microinjector and then cultured the E10.5 mouse embryos in the roller culture system for 24 h. We used untargeted siRNA tagged with the fluorescent dye, CY3 as control also referred to as siRNA CTL (siGLO- MWG Biotech), and to localize the site of injection of *foxd3* and *wnt1* siRNAs, we co-administered them with the siGLO. The silencing was monitored by RT-qPCR.

## Quantification and Statistical Analysis

### Imaging Analysis.

Imaging analysis and area for counting and for growth quantification were manually annotated. Limb regrowth was quantified as the area from the amputation plane to the most distal tip, ensuring consistency across all experiments, and measured using Fiji (29).

### Statistical Analysis.

Mann–Whitney, one tail was performed to test significance for Figs. 1, 3, and 4 and *SI Appendix*, Figs. S1 and S4 using GraphPad Prism 6 Software (San Diego, CA).

## Supplementary Material

Appendix 01 (PDF)

## Data Availability

All data are available in the Gene Expression Omnibus (GEO) repository (https://www.ncbi.nlm.nih.gov/geo/) under the provisional accession number GSE292836 (30). All other data are included in the manuscript and/or *SI Appendix*.
