# Supplementary material for: Neural crest cell recruitment and reprogramming as central drivers of embryonic limb regeneration
Source: Proc Natl Acad Sci U S A. 2025 Dec 23;122(52):e2519994122. doi: 10.1073/pnas.2519994122 (PMC12772167; doi:10.1073/pnas.2519994122)
Supplement: Supplementary file 1 — Appendix 01 (PDF) [file pnas.2519994122.sapp.pdf]

PNAS

Supporting Information for

Neural Crest Cell Recruitment and Reprogramming as Central Drivers of Embryonic Limb Regeneration

Béryl Laplace-Builhé , Gautier Tejedor , Jholy De La Cruz , Audrey Barthelaix , Frédéric Marmigère , Dora Sapède , Sarah Bahraoui , Lucie Diouloufet , Stéphanie Ventéo , Jérôme Collignon , Christian Jorgensen , Farida Djouad

Corresponding Author: Farida Djouad
Email: farida.djouad@inserm.fr

This PDF file includes:

Figures S1 to S4

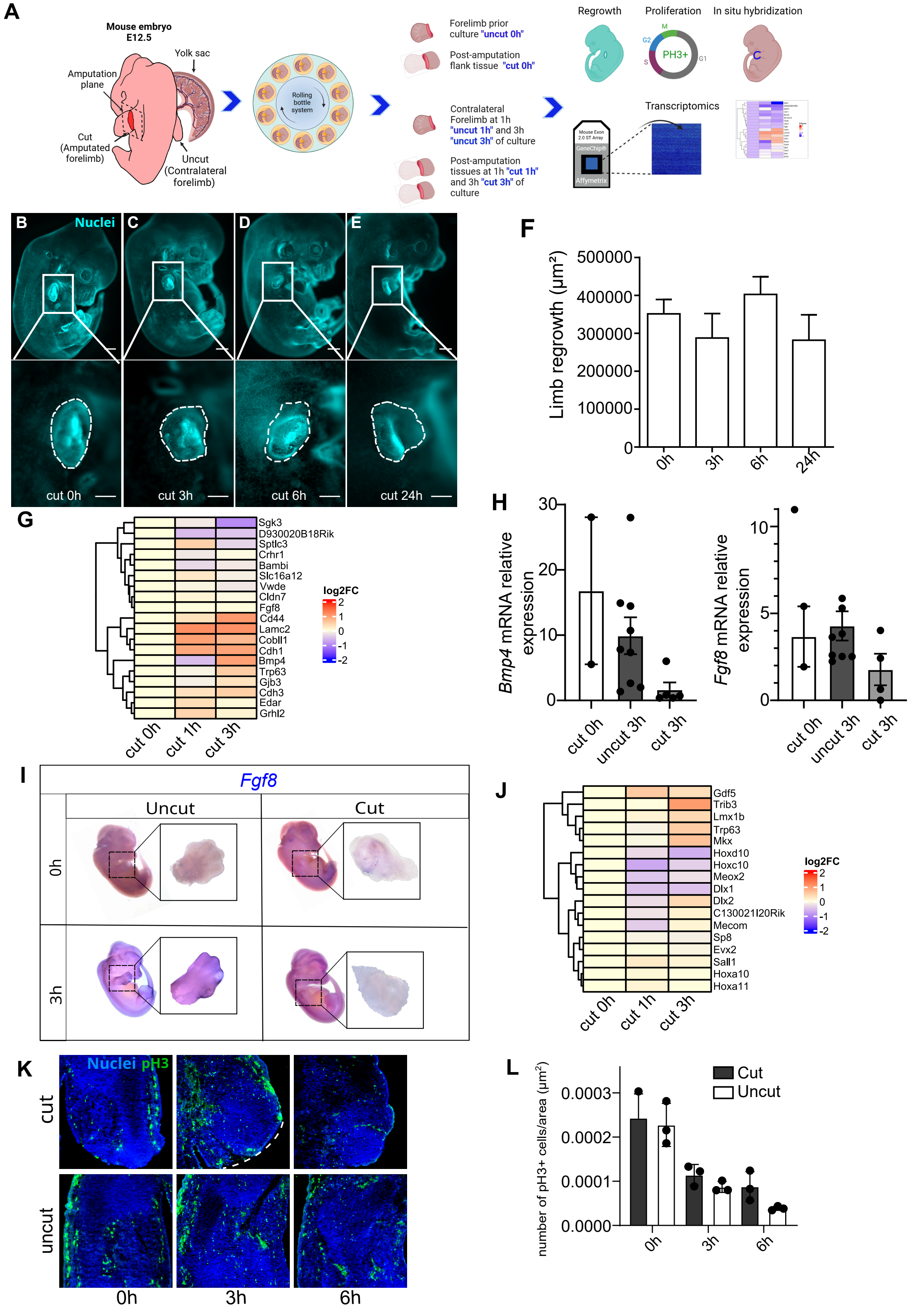
A
Mouse embryo
E12.5
Yolk sac
Amputation plane
Cut (Amputated forelimb)
Uncut (Contralateral forelimb)
Rolling bottle system
Forelimb prior culture "uncut 0h"
Post-amputation flank tissue "cut 0h"
Contralateral Forelimb at 1h "uncut 1h" and 3h "uncut 3h" of culture
Post-amputation tissues at 1h "cut 1h" and 3h "cut 3h" of culture
Regrowth
Proliferation
In situ hybridization
PH3+
Transcriptomics
B
Nuclei
C
D
E
cut 0h
cut 3h
cut 6h
cut 24h
F
Limb regrowth (µm²)
500000
400000
300000
200000
100000
0
0h
3h
6h
24h
G
Sgk3
D930020B18Rik
Sptlc3
Crhr1
Bambi
Slc16a12
Vwde
Cldn7
Fgf8
Cd44
Lamc2
Cobll1
Cdh1
Bmp4
Trp63
Gjb3
Cdh3
Edar
Grhl2
log2FC
cut 0h
cut 1h
cut 3h
H
Bmp4 mRNA relative expression
Fgf8 mRNA relative expression
cut 0h
uncut 3h
cut 3h
I
Fgf8
Uncut
Cut
0h
3h
J
Gdf5
Trib3
Lmx1b
Trp63
Mkx
Hoxd10
Hoxc10
Meox2
Dlx1
Dlx2
C130021I20Rik
Mecom
Sp8
Evx2
Sall1
Hoxa10
Hoxa11
log2FC
cut 0h
cut 1h
cut 3h
K
Nuclei pH3
cut
uncut
0h
3h
6h
L
number of pH3+ cells/area (µm²)
0.0003
0.0002
0.0001
0.0000
Cut
Uncut
0h
3h
6h

Fig. S1. Mouse embryos are not able to regenerate the forelimb at E12.5. (A) Workflow of the experiment and sample annotation. (B-E) Macroscopic fluorescent images of E12.5 embryos 24hours after amputation of the forelimb cultured for different times, fixed and nuclei labeled at different time. Left forelimb images were flipped to facilitate comparison (B) E12.5 at 0 hpA, (C) 3 hpA, (D) 6 hpA, 24 hpA (E) (for the upper panels=800µm, for the bottom panels Scale bars=400µm). Dashed lines delineate the area measured for quantification of regrowth. (F) Corresponding graph representing area quantification of remaining limb tissue E12.5 embryos (Graphs represent the mean, error bars are *SEM*, n=3-6 embryos per group). (G) Expression profile of AER markers in post-amputation tissues of E12.5 embryos at 0hpa (cut 0h) and in post-amputation tissues 1hpa (cut 1h) and 3hpa (cut 3h). (H) Relative expression of *Bmp4* and *Fgf8* mRNA levels assessed by RT-qPCR in the tissues surrounding the amputation area of amputated E12.5 mouse embryos (cut 0h), the FB of *ex utero* developing intact E12.5 mouse embryos (3 hours post-culture/ uncut 3h) and the tissue remaining after amputation of *ex utero* cultured E12.5 mouse embryos (3 hours post-amputation/ cut 3h). (I) *In situ* hybridization against *Fgf8* mRNA in E12.5 mouse embryos. All *in situ* hybridizations were performed in embryos before and after 3 hours of culture. (I) *Fgf8* expression in the intact non-cultured E12.5 embryo, and higher magnification of the dissected FB of non-cultured E12.5 embryo. *Fgf8* transcripts were not detected in forelimb. *Fgf8* expression in the amputated non cultured E12.5 embryo, and higher magnification of the dissected FB of amputated non cultured E12.5 embryo. *Fgf8* transcripts were not detected in the amputated area. *Fgf8* expression in intact E12.5 embryo 3 hours post-culture, and higher magnification of the dissected FB of intact E12.5 embryo 3 hours post-culture. *Fgf8* transcripts were not detected in forelimb. *Fgf8* expression in the amputated E12.5 embryo 3 hours post-culture with higher magnification of the dissected forelimb (J) Expression profile of distal mesenchymal markers from post-amputation tissues at 0hpa (cut 0h), 1 hpA (cut 1h) and 3 hpA (cut 3h) at E12.5 stage. (K) Proliferation was assessed by immunofluorescence using a pH3 antibody (green) on cryosections at 0h, 3h, 6h, post amputation of FB E12.5 counterstained with Hoechst (blue) (dashed line delineates the amputation site). (L) Graph represents quantification of proliferation in the forelimb cryosections at 0h, 3h, 6h and 24h after the amputation of E12.5 embryos. Proliferation was assessed by immunofluorescence using a pH3 antibody (Graphs represent means, error bars are *SEM*).

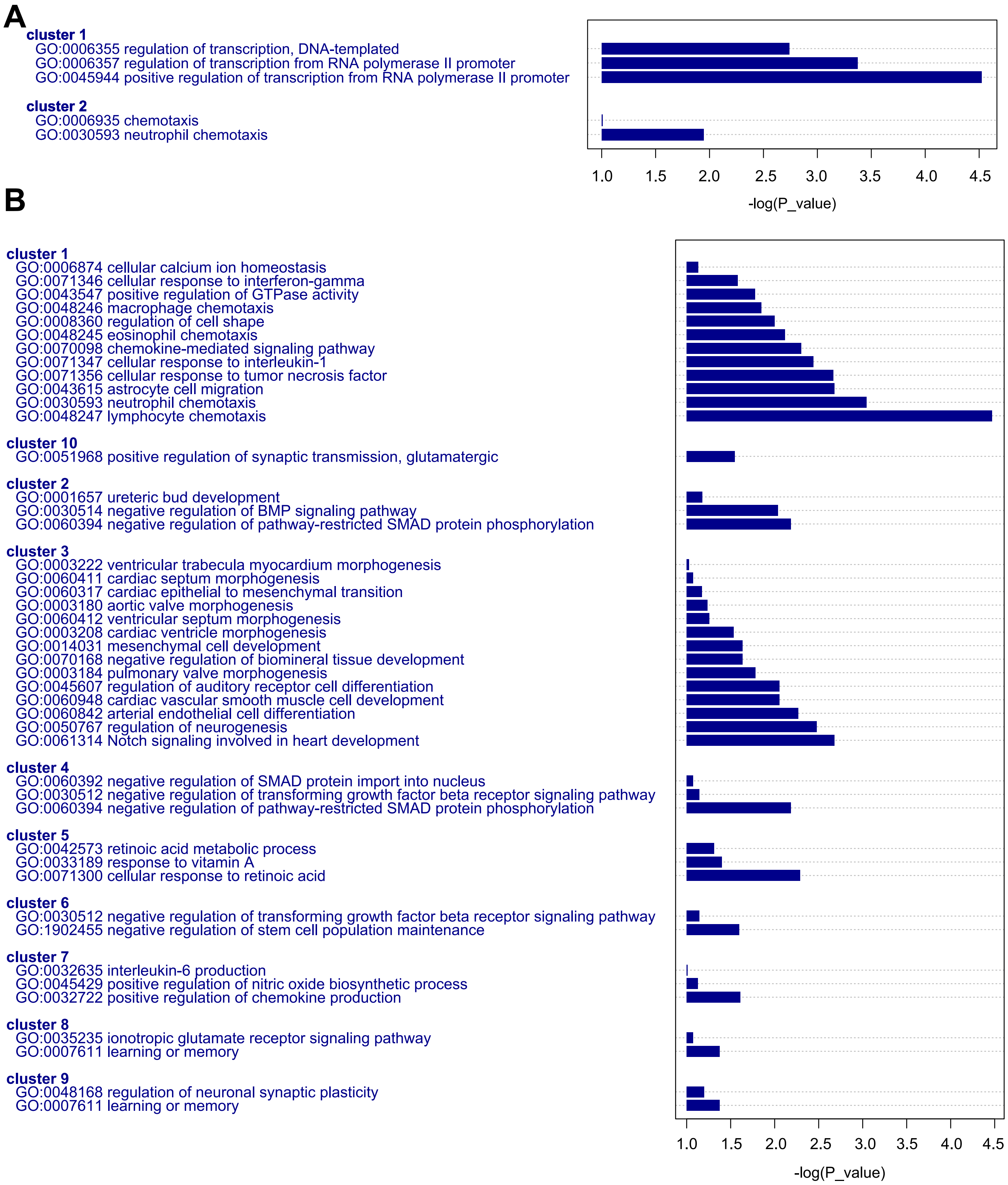
A
cluster 1
GO:0006355 regulation of transcription, DNA-templated
GO:0006357 regulation of transcription from RNA polymerase II promoter
GO:0045944 positive regulation of transcription from RNA polymerase II promoter
cluster 2
GO:0006935 chemotaxis
GO:0030593 neutrophil chemotaxis
1.0 1.5 2.0 2.5 3.0 3.5 4.0 4.5
-log(P_value)
B
cluster 1
GO:0006874 cellular calcium ion homeostasis
GO:0071346 cellular response to interferon-gamma
GO:0043547 positive regulation of GTPase activity
GO:0048246 macrophage chemotaxis
GO:0008360 regulation of cell shape
GO:0048245 eosinophil chemotaxis
GO:0070098 chemokine-mediated signaling pathway
GO:0071347 cellular response to interleukin-1
GO:0071356 cellular response to tumor necrosis factor
GO:0043615 astrocyte cell migration
GO:0030593 neutrophil chemotaxis
GO:0048247 lymphocyte chemotaxis
cluster 10
GO:0051968 positive regulation of synaptic transmission, glutamatergic
cluster 2
GO:0001657 ureteric bud development
GO:0030514 negative regulation of BMP signaling pathway
GO:0060394 negative regulation of pathway-restricted SMAD protein phosphorylation
cluster 3
GO:0003222 ventricular trabecula myocardium morphogenesis
GO:0060411 cardiac septum morphogenesis
GO:0060317 cardiac epithelial to mesenchymal transition
GO:0003180 aortic valve morphogenesis
GO:0060412 ventricular septum morphogenesis
GO:0003208 cardiac ventricle morphogenesis
GO:0014031 mesenchymal cell development
GO:0070168 negative regulation of biomineral tissue development
GO:0003184 pulmonary valve morphogenesis
GO:0045607 regulation of auditory receptor cell differentiation
GO:0060948 cardiac vascular smooth muscle cell development
GO:0060842 arterial endothelial cell differentiation
GO:0050767 regulation of neurogenesis
GO:0061314 Notch signaling involved in heart development
cluster 4
GO:0060392 negative regulation of SMAD protein import into nucleus
GO:0030512 negative regulation of transforming growth factor beta receptor signaling pathway
GO:0060394 negative regulation of pathway-restricted SMAD protein phosphorylation
cluster 5
GO:0042573 retinoic acid metabolic process
GO:0033189 response to vitamin A
GO:0071300 cellular response to retinoic acid
cluster 6
GO:0030512 negative regulation of transforming growth factor beta receptor signaling pathway
GO:1902455 negative regulation of stem cell population maintenance
cluster 7
GO:0032635 interleukin-6 production
GO:0045429 positive regulation of nitric oxide biosynthetic process
GO:0032722 positive regulation of chemokine production
cluster 8
GO:0035235 ionotropic glutamate receptor signaling pathway
GO:0007611 learning or memory
cluster 9
GO:0048168 regulation of neuronal synaptic plasticity
GO:0007611 learning or memory
1.0 1.5 2.0 2.5 3.0 3.5 4.0 4.5
-log(P_value)

Fig. S2. (A) Functional clustering of gene ontology (GO) terms enriched in differentially expressed genes (DEGs) following forelimb bud (FB) amputation in both E10.5 and E12.5 mouse embryos revealed two shared clusters of GO terms: Cluster 1 and Cluster 2. (B) Functional clustering of GO terms specifically enriched in DEGs from E12.5 post-amputation tissues.

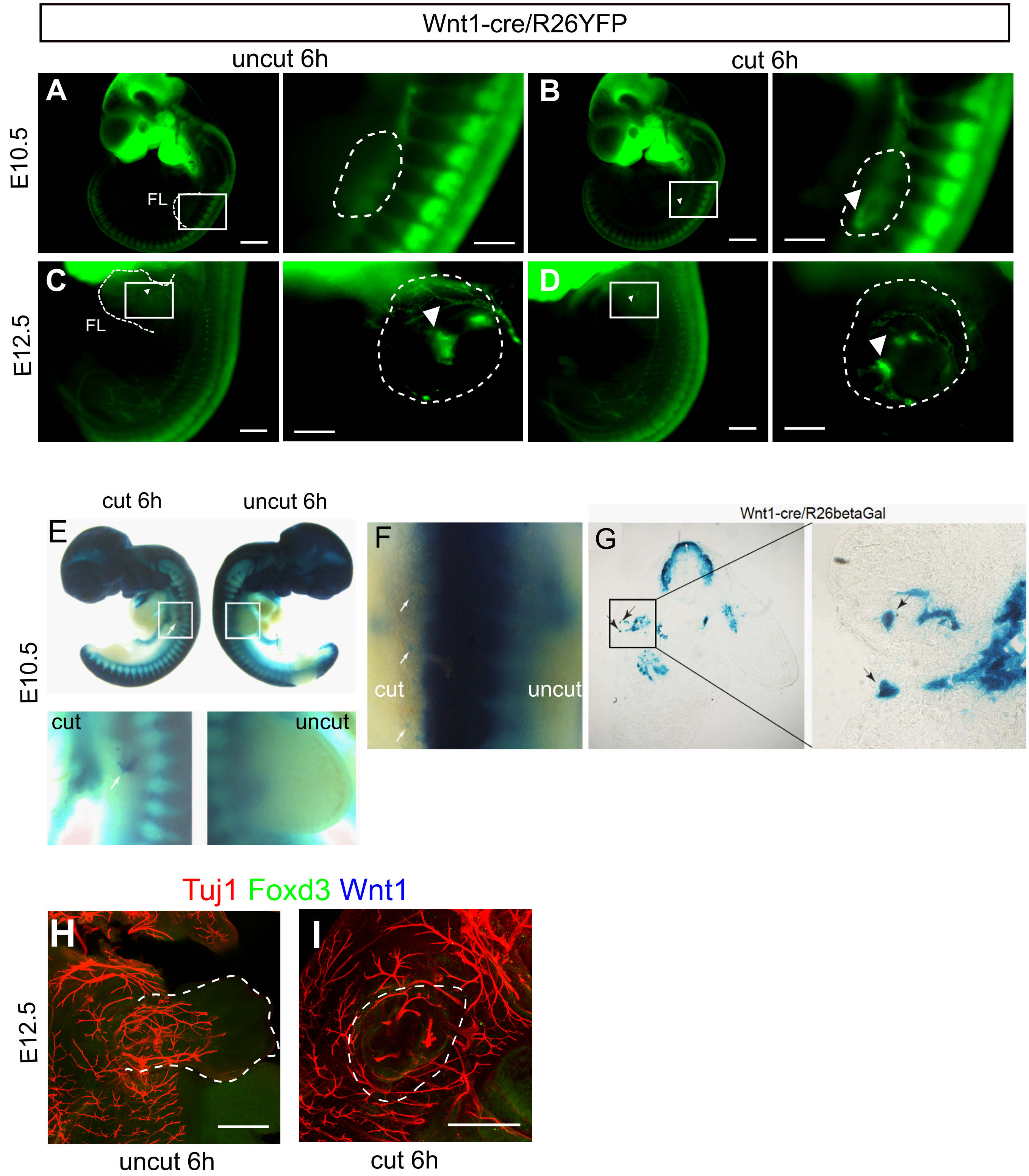

Wnt1-cre/R26YFP
uncut 6h
cut 6h
A
B
C
D
E10.5
E12.5
FL
cut 6h
uncut 6h
E
F
G
Wnt1-cre/R26betaGal
cut
uncut
Tuj1 Foxd3 Wnt1
H
I
uncut 6h
cut 6h

Fig. S3. (A) and (B) Whole mount staining images of E10.5 (upper panels, n=15), (C, D) Whole mount staining images of E12.5 (bottom panels, n= 9) from the Wnt1-cre/R26YFP at 6hpA, showing amputated (cut) or contralateral (uncut) limb as indicated. Dashed lines delineate the post-amputation injured areas and the equivalent areas in the controlateral forelimbs (FL). NC-derived cells are green (indicated by white arrowheads). (A, B, C, and D) have scale bars of 400 µm on the left and 800 µm on the right, respectively. (E) Images of Wnt1-cre/R26βGal E10.5 embryos at 6 hours post amputation after X-Gal staining (white arrows indicate NCC in blue). (F) Dorsal view picture of a Wnt1-cre/ R26βGal E10.5 embryo at the level of the forelimb, 6 hours post-amputation. Arrows point at β-Gal-positive cells located distal to the DRG. (G) Images of Wnt1-cre/R26βGal E10.5 embryo transversal cryosections at 6 hours post-amputation. Black arrows indicate NCC (in blue). (H), (I) Images from confocal microscopy of cleared E12.5 embryos labeled with TUJ1 (red), WNT1 (blue) and FOXD3 (green) antibody, showing contralateral intact FB (H), and amputated FB at 6 hours post-amputation (I) (Scale bars = 400µm).

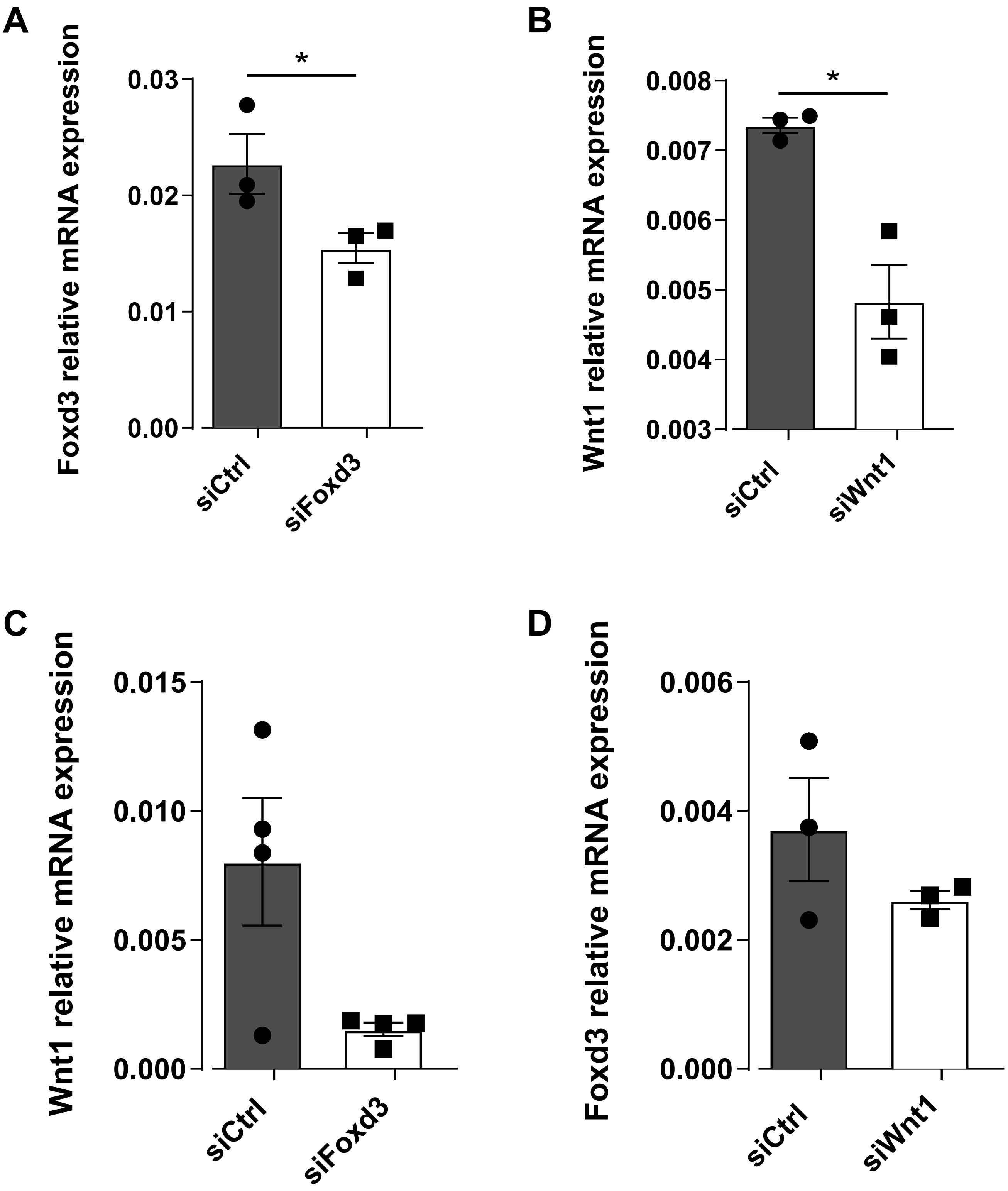

A
Foxd3 relative mRNA expression
0.03
0.02
0.01
0.00
*
siCtrl
siFoxd3
B
Wnt1 relative mRNA expression
0.008
0.007
0.006
0.005
0.004
0.003
*
siCtrl
siWnt1
C
Wnt1 relative mRNA expression
0.015
0.010
0.005
0.000
siCtrl
siFoxd3
D
Foxd3 relative mRNA expression
0.006
0.004
0.002
0.000
siCtrl
siWnt1

Fig. S4. (A), (D) Foxd3 mRNA relative expression in siRNA control or siRNA anti-Foxd3 or siRNA anti-Wnt1 transfected E10.5 embryo forelimbs. (B), (C) Wnt1 mRNA relative expression in siRNA control or siRNA anti-Foxd3 or siRNA anti-Wnt1 transfected E10.5 embryo forelimbs. RT-qPCR were performed using Rps9 as a reference gene (error bars are *SEM*, n=3-4 embryos per group, *p<0.05).
